# Quorum sensing improves the plant growth-promoting ability of *Stenotrophomonas rhizophila* under saline-alkaline stress by enhancing its environmental adaptability

**DOI:** 10.3389/fmicb.2023.1155081

**Published:** 2023-04-11

**Authors:** Xuliang Zhuang, Ying Liu, Na Fang, Zhihui Bai, Jie Gao

**Affiliations:** ^1^CAS Key Laboratory of Environmental Biotechnology, Research Center for Eco-Environmental Sciences, Chinese Academy of Sciences, Beijing, China; ^2^College of Resources and Environment, University of Chinese Academy of Sciences, Beijing, China; ^3^State Key Laboratory of Tibetan Plateau Earth System, Environment and Resources (TPESER), Institute of Tibetan Plateau Research, Chinese Academy of Sciences, Beijing, China; ^4^Institute of Advanced Agricultural Sciences, Peking University, Weifang, China; ^5^Institute of International Rivers and Eco-security, Yunnan University, Kunming, China; ^6^Xiong’an Institute of Innovation, Xiong’an New Area, China

**Keywords:** quorum sensing (QS), diffusible signal factor (DSF), *Stenotrophomonas rhizophila*, saline-alkaline stress, *Brassica napus* L.

## Abstract

Quorum sensing (QS) system has an essential function in plant growth-promoting rhizobacteria (PGPR) response to environmental stress and PGPR induction of plant tolerance to saline-alkaline stress. Nevertheless, there is a lack of understanding about how QS influences the growth-promoting effects of PGPR on plants. *Stenotrophomonas rhizophila* DSM14405^T^ is a PGPR with a QS system, which can secrete diffusible signal factor (DSF), one of the QS signal molecules. In this study, we used the *S. rhizophila* wild type (WT) and an incompetent DSF production *rpfF*-knockout mutant strain to explore whether DSF-QS could affect the growth-promoting ability of PGPR in *Brassica napus* L. By measuring the seed germination rate, plant fresh weight, biomass, the total antioxidant capacity (T-AOC) level, and the content of chlorophyll in leaves, we found that DSF was unable to enhance the growth-promoting capacity of Δ*rpfF* and did not directly assist the plants in tolerating saline-alkaline stress. However, DSF aided *S. rhizophila* Δ*rpfF* in resisting stress during its effective period, and QS represents a continuous and precise regulatory mechanism. Altogether, our results show that DSF is helpful to improve the environmental adaptability and survival rate of *S. rhizophila*, thus indirectly improving the germination rate of seeds and helping plants grow under saline-alkaline stress. In this study, the mechanism of QS enhancing the environmental adaptability of PGPR was studied, which provided a theoretical basis for improving the application of PGPR to help plants better cope with saline-alkaline stress.

## 1. Introduction

Saline-alkaline stress seriously impacts plant growth and development (Xu et al., 2021). Soil salinization and alkalinization often co-occur, devastatingly impacting plant growth (Liu et al., 2020). Plants must contend with ionic toxicity, osmotic stress, and high pH stress concurrently in saline-alkaline environments (Li et al., 2016).

Rhizosphere microbes play a vital role in helping plant species adjust to saline and alkaline stress (Lian et al., 2020). In particular, plant-growth promoting rhizobacteria (PGPR) are generally used as microbial fertilizers due to their noteworthy growth promotion and salinity resistance effects (Paul and Lade, 2014; Kumar and Verma, 2018). *Stenotrophomonas rhizophila* DSM14405^T^ is a non-pathogenic, rhizosphere- and phylloplane-dwelling bacterium that shows pronounced saline tolerance by producing highly effective osmoprotectants, such as glucosyl glycerol (GG), trehalose, and spermidine, which act directly upon roots and aid plant survival in harsh environmental conditions (Alavi et al., 2013b; Egamberdieva et al., 2016). *S. rhizophila* is positioned within the family *Xanthomonadaceae* and has *rpf*-gene clusters (Huedo et al., 2015), a gene cluster encoding diffusible signal factor (DSF)- in gram-negative bacteria.

The quorum sensing (QS) signal molecules of gram-negative bacteria are mostly composed of acyl homoserine lactones, autoinducer-2, and DSF. DSF, whose chemical formula is *cis*-11-methyl-2-dodecenoic acid (Wang et al., 2004), is a member of the unsaturated fatty acid family. DSF is mainly produced in *Xanthomonadaceae* taxon under condition of high population density. QS is the key mechanism for PGPR to regulate bacterial population density, metabolic activity, and interaction, and controls multiple physiological behaviors of cells (Calatrava-Morales et al., 2018; Phour et al., 2020). QS enables bacteria to coordinate gene expression according to local population density. During the process of bacterial proliferation, the synthesis of QS signal molecules is secreted to the extracellular, and the concentration of the extracellular signal molecules increases with the increase of population density. When the concentration of the QS signal molecules reaches a certain threshold, it is induced by the corresponding receptor protein of the bacteria to start the related gene expression and protein synthesis, and carry out metabolic regulation function. When the physiological activity of bacteria is completed, bacteria exit QS (Zhou et al., 2017b). In Gram-negative bacteria, the *rpf* gene cluster is responsible for the production and perception of DSF: the *rpfF* encodes DSF synthase RpfF, *rpfB* encodes fatty acyl-CoA ligase RpfB. The histidine kinase sensor and response regulator encoded by *rpfC* and *rpfG*, respectively, constitute a two-component regulatory system (Ryan et al., 2015; Zhou et al., 2017b).

In addition, Alavi et al. (2013a) showed that DSF contributes to host colonization by the PGPR *Stenotrophomonas maltophilia* and an improved seed germination rate and plant growth promotion. Our previous studies have demonstrated that DSF-QS in *S. rhizophila* DSM14405^T^ can help bacteria resist saline-alkaline stress by regulating metabolic activities (Liu et al., 2022). Hence, we want to further address the role of DSF in affecting plant growth promotion in *S. rhizophila*.

Here, we use *S. rhizophila* and oilseed rape (*Brassica napus* L.), investigating the impact of DSF-QS on the germination and growth of plants under conditions of saline-alkaline stress. The dynamics of bacteria-plant signaling related to QS warrant further investigation and should be applied in PGPR techniques to help crops cope with saline-alkaline stress with the goal of promoting sustainable agriculture.

## 2. Materials and methods

### 2.1. Bioinformatic analyses

Diffusible signal factor synthase encoded by *rpfF*, is crucial for the product of DSF (Alavi et al., 2014). To identify and recognize the gene encoding DSF synthases in *S. rhizophila*, the RpfF protein sequences were analyzed using the NCBI BLASTp program^1^. The DSF synthase sequences of selected species in the *Xanthomonadaceae* family were found in the NCBI protein database^2^. Multiple amino acid sequence alignments were generated by Clustal Omega^3^ and visualized in Jalview software (v2.11.1.4).

### 2.2. Construction of the *S. rhizophila rpfF* mutant

The total DNA of *Stenotrophomonas rhizophila* strain DSM14405^T^ was extracted by the TIANamp Bacteria DNA Kit (TIANGEN, Beijing, China). Primers *rpfF*-MF1/*rpfF*-MR1 and *rpfF*-MF2/*rpfF*-MR2 (Supplementary Table 1) were used to amplify the upstream and downstream homologous arm fragments of gene *rpfF*, respectively. Homologous arm fragments *rpfF*-MF1 and *rpfF*-MR2 were purified by gel electrophoresis, then used as templates for overlapping PCR amplification. The purified overlap PCR fragment was seamlessly connected to the suicide vector pLP12 by applying recombinant Exnase II (ClonExpress II; Vazyme) at 37°C for 30 min, and the resulting plasmid was transformed into *Escherichia coli* DH5α competent cells (TIANGEN, Beijing, China). Transformed bacteria were spread on LB plates containing 20μg/mL chloramphenicol and 0.3% d-glucose.

Primers *rpfF*-UF and *rpfF*-UR were used to screen recombinant clones carrying the homologous arm fragments. A positive clone was cultured, plasmid plP12-*rpfF* was extracted, and transformed into *E. coli* β2163. *E. coli* β2163 (carrying recombinant plasmid pLP12-*rpfF*) and *S. rhizophila* were cultured overnight, respectively. A 100 and 150 μL sample of each bacterial culture were mixed, centrifuged, and resuspended by LB medium twice. The cell pellet mixture was resuspended in 10μL LB medium, spread on an LB plate containing 0.3 mM diaminoheptanoic acid and 0.3% d-glucose cultured at 30°C for 20 h. Cells were then resuspended in 1 mL LB medium, streaked on an LB plate containing 20μg/ml chloramphenicol and 0.3% d-glucose.

The *E. coli* β2163 donor strain could not grow on the LB plate containing 20μg/mL chloramphenicol and 0.3% d-glucose; only *S. rhizophila* cells harboring the plasmid inserted into the chromosome finger site *via* homologous recombination could survive.

The resulting clones were cultured in LB medium and confirmed using primers *rpfF*-MF1 and *rpfF*-MR2. The clones were then spread on an LB plate containing 0.4% L-arabinose and cultured overnight at 30°C. Only when the second homologous recombination event occurred and suicide plasmid pLP12 was lost could bacteria survive. Primers *rpfF*-TF and *rpfF*-TR were used for PCR screening of colonies, and wild-type, WT (1970 bp), and knock-out mutant strain (1298bp) were distinguished.

### 2.3. DSF extraction and detection

The QS signal DSF was extracted according to the procedure described by He et al. (2010), albeit with minor modifications. The *S. rhizophila* WT and *rpfF* mutant strains were, respectively, cultured on standard LB medium (1% NaCl, pH 7) and 2% NaCl LB medium, at pH 9, for 24, 48, 72 h, respectively, and the absorbance (OD 600 nm) was determined. The bacterial supernatant (500 mL) was collected by centrifugation at 4000 × *g* for 30 min at 4°C. The pH of these supernatants was adjusted to 3.0–4.0, by adding hydrochloric acid prior to extraction with an equal volume of ethyl acetate twice. The ethyl acetate fractions were then collected and the solvent was removed, by rotary evaporation at 40 to dryness. The extracts were dissolved in 1 mL of acetonitrile and filtered through 0.22-μm filters into UHPLC vials and stored at −20°C until their analysis. The experiment was repeated three times.

Diffusible signal factor standard was purchased from Sigma (St. Louis, MO, USA), and the calibration curves included calibrators at 0, 0.5, 1, 5, 10, 20, 50, 100, 200 μg/L DSF in acetonitrile. 450 μL Samples were taken out and added 50 μL DSF (15 mg/L) was as spiked samples. Standards, samples, and spiked samples were loaded onto a SIL-30AC autosampler set at 10°C. 5 μL of each standard and sample was injected onto a 2 um Shim-pack GIST C18 2.1 mm x 100 mm column (SHIMADZU, Japan) at 35°C using reversed-phase chromatography. Mobile phase A was 5% solution buffer in water; mobile phase B was 5% (ν/ν) buffer solution, 55% (ν/ν) acetonitrile, and 40% (ν/ν) isopropanol. The buffer solution used was 100 mM glacial acetic acid, and the pH was adjusted to 5 using ammonium hydroxide. The LC time gradient was created using LC-30AD detecting system (Shimadzu) as follows: 0–6 min, 70–100% B; 6–8 min, 100% B; 8–10 min, 100–70% B. The entire gradient was run at a flow rate of 0.3 mL/min.

Heated electrospray ionization in negative modes with multiple reaction monitoring (MRM) on a Shimadzu LCMS-8050 triple-quadrupole mass spectrometer was used to detect DSF. Because of the non-ideal fragmentation behavior of fatty acids, we used “pseudo-molecular” MRM with optimized collision cell parameters (Schiesel et al., 2010), choosing the same ions as the precursor and product ions: the precursor was 211.1 *m/z*, and the product was 211.1 *m/z*. The MS/MS conditions for DSF were optimized using the automated MRM optimization procedure in LabSolutions (Shimadzu). The interface temperature was 300°C, desolvation line temperature was 250°C, and heat block temperature was 400°C. Nebulizing gas flow was 3 L/min, heating gas flow was 10 L/min, drying gas flow was 10 L/min, dwelltime is 20 ms, Q1 pre bias is 15 V, CE is 7 V, Q3 pre bias is 24 V.

### 2.4. DSF addback experiment

*S. rhizophila* WT and Δ*rpfF* were, respectively, cultured on standard LB medium at 30°C with shaking at 150 rpm, until the OD 600 nm value of the cells reached about 1.1. The bacterial cultures were, respectively, collected by centrifugation at 4000 × *g* for 30 min at 4°C, the supernatant was discarded and the cells were, respectively, resuspended in the same volume of 2% NaCl LB medium, at pH 9. As shown in Table 1, four experimental groups were set up: WT and Δ *rpfF* groups were not treated; Δ *rpfF* add DSF once and Δ *rpfF* add DSF twice groups were, respectively, added DSF in the initial state, to make its final concentration of DSF in the cultures was 35 μg/L. Each experimental group has six parallels at 30°C with shaking at 150 rpm, and the cell density was detected during the growth process. After 14 h, DSF was added to the Δ *rpfF* add DSF twice group again to make its final concentration of DSF in the culture 1333 μg/L, and the other experimental groups were not treated. Continue to culture the groups at 30°C with shaking at 150 rpm, and detect the cell density at the 16th hour.

**TABLE 1:** DSF addback experiment.

Group	WT	Δ*rpfF*	Δ*rpfF* add DSF once	Δ*rpfF* add DSF twice
Strain	*S. rhizophila* WT	*S. rhizophila* Δ*rpfF*	*S. rhizophila* Δ*rpfF*	*S. rhizophila* Δ*rpfF*
Additional DSF	/	/	Initial 0 h add DSF	Initial 0 h add DSF, 14 h add DSF

### 2.5. Seed germination under saline-alkaline stress

*Brassica napus* oilseeds were first surface-sterilized in 2% NaClO for 10 min and then washed with sterile water three times. Take 200 μL water from the third rinse and spread it onto LB plates at 30°C overnight to check for contamination. Continue with the following steps if there is no microbial colony presenting on the LB plates, otherwise, discard the seeds and repeat disinfection of seeds until no colony is detected on the LB plates. The seeds were placed in sterile gauze, and divided into eight groups, each group had three parallels, and each parallel had one hundred seeds for different treatments (Table 2). The Group CKN was treated with sterile water, and the other seven groups were treated with sterile water containing 2% NaCl and pH 9: Group CK: no operation; Group W: *S. rhizophila* WT; Group F: *S. rhizophila* Δ*rpfF*; Group SW: autoclaved *S. rhizophila* WT; Group SF: autoclaved *S. rhizophila* Δ*rpfF*; Group D: DSF and Group F + D: *S. rhizophila* Δ*rpfF* + DSF. The number of bacteria per plate for *S. rhizophila* WT, Δ*rpfF*, and inactivated strains in W, F, SW, and SF groups were 3 × 10^9^, respectively. The amount of DSF added to each plate in D and D + F groups was 10 μg. Plants were arranged in a replicated randomized block design, maintained at 20°C under a 12 h/12 h light/dark photoperiod, and grown for 5 days. Determine the germination rate of the plant.

**TABLE 2:** Experiment on seed germination under saline-alkaline stress.

Group	CKN	CK	W	F	SW	SF	D	F + D
Treatment	/	/	*S. rhizophila* WT	*S. rhizophila* Δ*rpfF*	Autoclaved *S. rhizophila* WT	Autoclaved *S. rhizophila* Δ*rpfF*	DSF	*S. rhizophila* Δ*rpfF* + DSF
Added substance	Sterile water, pH 7	2% NaCl sterile water, pH 9	2% NaCl sterile water, pH 9	2% NaCl sterile water, pH 9	2% NaCl sterile water, pH 9	2% NaCl sterile water, pH 9	2% NaCl sterile water, pH 9	2% NaCl sterile water, pH 9

We washed the gauze in groups W and F with 100 mL sterile water three times, mixed the eluent, then diluted the eluent in a gradient, applied it to the LB plate, and calculated the CFU of bacteria to count the active *S. rhizophila* WT and Δ*rpfF* in each plate. In addition, monoclone were randomly selected for strain identification to ensure that the bacteria were *S. rhizophila* rather than miscellaneous bacteria.

### 2.6. Plant potting under saline-alkaline stress

Disinfection and germination of seeds are described above, plant seedlings were then planted into sterilized vermiculite, which was sterilized twice *via* autoclaving at 121°C for 20min each. Plants were arranged in a replicated randomized block design and maintained at 20°C under a 12 h/12 h light/dark photoperiod and sustained with standard Hoagland solution (pH 6). After reaching the fourth-leaf stage, plants showing uniform growth were randomly divided into eight groups, transferred to soil for growth (see Supplementary Table 2 for soil properties), and divided into CKN, CK, W, F, SW, SF, D, and F + D groups according to the grouping treatment described in Table 3. The following substances were added to each group of soil for treatment: Group W (*S. rhizophila* WT bacterial liquid, 2 × 10^8^ CFU per gram of soil); Group F (Δ*rpfF* bacterial suspension, 2 × 10^8^ CFU per gram of soil); Group SW (same amount of WT after autoclaving); Group SF (same amount of Δ*rpfF* after autoclaving); Group D (10 μg DSF per gram soil); Group F + D (2 × 10^8^ Δ*rpfF* + 10 μg DSF per gram soil); Group CK (equivalent LB medium); Group CKN (equivalent LB medium).

**TABLE 3:** Experiment on plant growth under saline-alkaline stress.

Group	CKN	CK	W	F	SW	SF	D	F + D
Treatment	/	/	*S. rhizophila* WT	*S. rhizophila* Δ*rpfF*	Autoclaved *S. rhizophila* WT	Autoclaved *S. rhizophila* Δ*rpfF*	DSF	*S. rhizophila* Δ*rpfF* + DSF
Added substance	Hoagland medium, pH 6	2% NaCl Hoagland medium, pH 9	2% NaCl Hoagland medium, pH 9	2% NaCl Hoagland medium, pH 9	2% NaCl Hoagland medium, pH 9	2% NaCl Hoagland medium, pH 9	2% NaCl Hoagland medium, pH 9	2% NaCl Hoagland medium, pH 9

The plants were transplanted into soil pots and grown in a light incubator at 20°C with a photoperiod of 12 h light/12 h dark. Every 72 h, 100 mL Hoagland medium (pH 6) was added to the Group CKN, and 100 mL 2% NaCl Hoagland medium (pH 9) was added to the other treatment groups. The experimental period was 5 weeks.

The newly grown and fully expanded leaves of the above-ground part of the plant were selected, and the chlorophyll content was quantified with SPAD-502 plus meter (Minolta Camera, Osaka, Japan) (Hu et al., 2018). To determine the total antioxidant capacity (T-AOC), the Fe^3+^ reduction method was applied using the T-AOC reagent kit (Solaibio, Beijing, China). The remaining aerial parts of the plants were then weighed fresh with a scale, and the plants were then dried at 60°C for 2 days to constant weight and weighed dry, and the dry weight was weighed as biomass.

## 3. Results

### 3.1. Greater production of DSF in *S. rhizophila* DSM14405^T^ WT under the saline-alkaline stress condition

**FIGURE 1:**
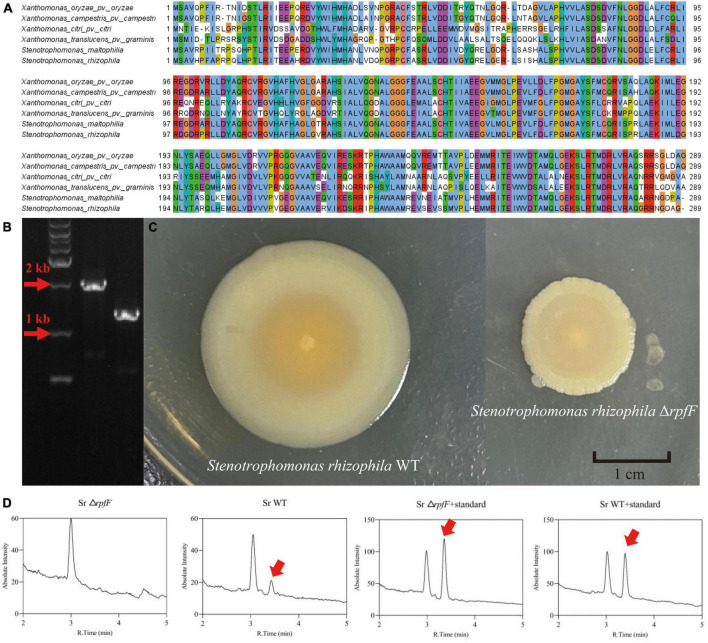
**(A)** Jalview-visualization of the multiple sequence alignment of the RpfF amino acid sequences in the selected repertoire of bacterial species. **(B)** Agarose gel validating the knockout of the *rpfF* gene, DNA maker (lane 1), *S. rhizophila* WT (lane 2), *S. rhizophila* Δ*rpfF* (lane 3). **(C)** Colony morphology of *S. rhizophila* WT and Δ*rpfF* strains grown on LB solid medium for 1 week. **(D)** Representative MRM (multiple-reaction monitoring) chromatogram of the *S. rhizophila* WT and Δ*rpfF* samples and corresponding DSF standards; the arrows point to the DSF peak.

Multiple amino acid sequence alignments result showed that the RpfF protein in *S. rhizophila* was highly (53.6–90.2%) similar to the DSF synthase in other bacteria known to produce the DSF signal (Figure 1A). Analysis of the RpfF amino acid sequence showed it was 77.6% identical to *Xanthomonas oryzae* pv. *oryzae* (He et al., 2010), 78.6% identical to *X. campestris* pv. *campestri* (Zhou et al., 2017b), 53.6% identical to *X. citri* pv. *citri* (Li et al., 2019), 54.5% identical to *X*. *translucens* pv. *graminis*, and 90.2% similar to *S. maltophilia* (Huang and Lee Wong, 2007). These alignments indicated that *S. rhizophila* is capable of synthesizing DSF.

The *rpfF* gene was knocked out by homologous recombination and we obtained the *rpfF* knockdown mutant, Δ*rpfF* (Figures 1B, C). The DSF was detected using a UHPLC-MS/MS quantitation analysis with MRM in the negative mode (Supplementary Figure 1), for which excellent linearity was observed within the calibration range (*R*^2^ > 0.999). The retention time was 3.48 min, with an accuracy ranging from 89.9 to 139.5%. The DSF detection of *S. rhizophila* WT and Δ*rpfF* samples, as well as the spiked samples, is depicted in Figure 1D. Evidently, the *S. rhizophila* WT can produce DSF, whereas the *rpfF* gene mutant strain cannot.

**FIGURE 2:**
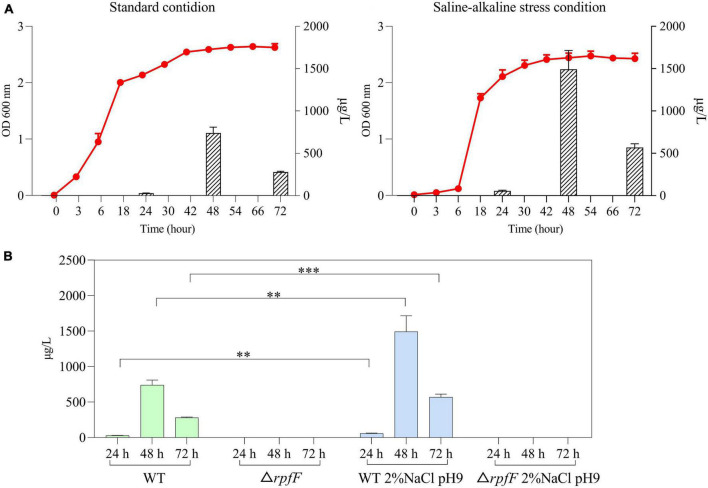
**(A)** Growth and DSF secretion of *S. rhizophila* WT under standard condition and saline-alkaline stress. The red curve represents the bacterial growth curve, and the bar graph represents the amount of DSF secretion. Error bars indicate ± SD, *n* = 3. **(B)** Detection of DSF in *S. rhizophila* WT and Δ*rpfF* under standard conditions and saline-alkaline stress. Error bars indicate ± SD, *n* = 3, **P < 0.01, ***P < 0.001 (independent sample *t*-test).

Under standard LB medium conditions, *S. rhizophila* WT secreted 28.6, 741.1, and 280.0 μg/L of DSF at 24, 48, and 72 h, respectively; when cultured on LB with 2% NaCl (pH 9) the *S. rhizophila* WT produced 56.3, 1492.6, and 569.1 μg/L of DSF at 24 h, 48 h, and 72 h, respectively. Cell densities were not significantly different between standard and saline-alkaline stress conditions (Figure 2A). The saline-alkaline stress dose significantly increased the production of DSF by around twofold (Figure 2B). The *S. rhizophila* Δ*rpfF* did not produce DSF in any of the cases. These results corroborate our previous study (Liu et al., 2022) and demonstrate that DSF-QS is a stress-responsive mechanism in *S. rhizophila.*

### 3.2. The role of DSF is time-sensitive

**FIGURE 3:**
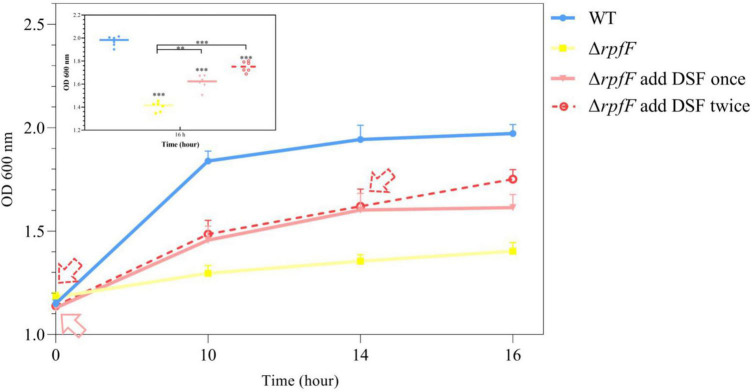
Effect of supplementation of DSF on the growth of Δ*rpfF*. The solid red arrow is the time point of a single addition of DSF; the red dashed arrow is the time point of two additions of DSF. The asterisks not marked with contrast lines represents a significant difference between each group and the group WT. Error bars indicate ± SD, *n* = 3. ANOVA, Tukey’s HSD test (**P < 0.01, ***P < 0.001).

Through the experiment of DSF supplementation, it was found that exogenous DSF could significantly increase the population density of *S*. *rhizophila* Δ*rpfF* under saline-alkaline stress and promote the growth of bacteria. As shown in Figure 3, comparing the growth of bacteria in the three experimental groups of Δ *rpfF*, Δ *rpfF* add DSF once, and Δ *rpfF* add DSF twice, noticeable that the addition of DSF promoted the growth of *S*. *rhizophila* Δ*rpfF*. At the same time, in the Δ *rpfF* add DSF twice group, the cell density increased significantly again with the second addition of DSF. This study found that the biological effect of exogenous DSF was time-limited, and it was gradually used and degraded in the process of bacterial growth and metabolism, and it needed to be added continuously to play its role. Exogenous addition of DSF could not increase the cell density of Δ*rpfF* to a level close to that of WT. Even if the secretion of DSF in *S. rhizophila* WT was known, when the corresponding concentration of DSF was added to Δ*rpfF*, the growth state of Δ*rpfF* could not be restored to the level at which QS is present.

### 3.3. Seed germination

**FIGURE 4:**
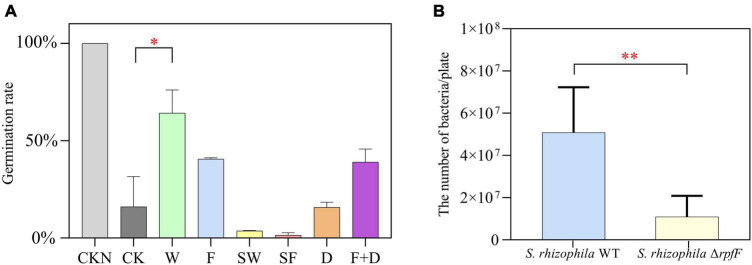
**(A)** Comparison of germination percentage of CKN, CK, W, F, SW, SF, and D groups, bar. Error bars indicate ± SD, *n* = 9 (ANOVA, followed by the Tukey’s HSD test, *P < 0.05). **(B)** Comparison of bacterial counts between W and F groups. Error bars indicate ± SD, *n* = 3 (Welch’s *t* test, **P < 0.01).

To study the role of QS in seed germination promoted by *S. rhizophila*, DSF, *S. rhizophila* WT, Δ*rpfF*, and both autoclaved strains were applied to plant seeds. The germination rates of CKN, CK, W, F, SW, SF, D and F + D groups were 100, 16.1, 64.3, 40.65, 3.7, 1.5, 15.8, and 39%, respectively. Compared with CKN and CK groups, saline-alkaline stress significantly inhibited the germination of seeds. Under stress, as shown in Figure 4A, *S. rhizophila* WT significantly increased the germination rate of the seeds by 298.7% compared with the CK group (*P* < 0.05). Compared with CK group, F and F + D groups also increased the mean of seed germination, but there was no significant difference, and the germination percentage was not improved in SW, SF, and D groups.

In addition, by measuring the active bacteria in W and F groups, we found that the number of *S. rhizophila* WT was significantly higher than the number of Δ*rpfF* after 5 days in the plates (Figure 4B).

### 3.4. Salt tolerance of plants

**FIGURE 5:**
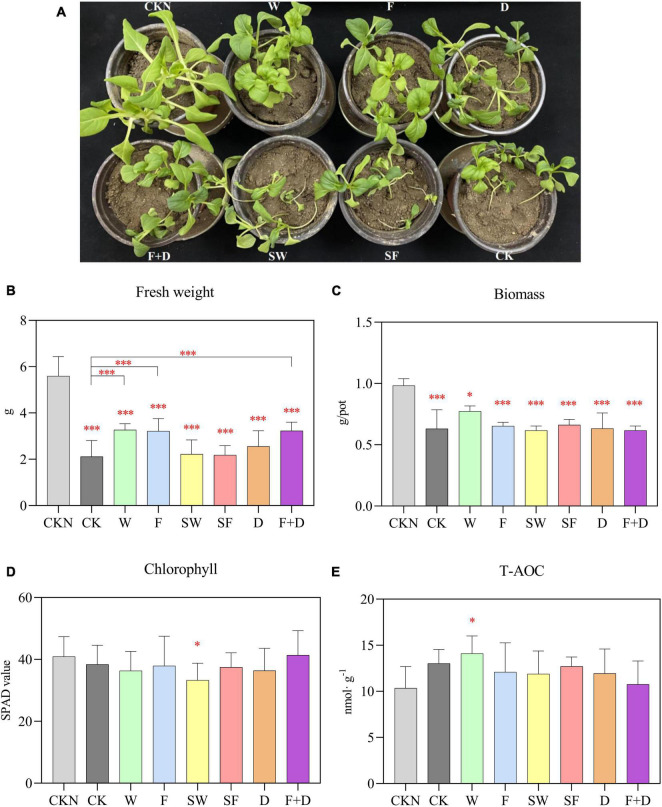
**(A)** Plant growth phenotype. Differences in fresh weight **(B)**, biomass **(C)**, chlorophyll **(D)**, and T-AOC **(E)** among CKN, CK, W, F, SW, SF, D, and F + D groups. The asterisks not marked with contrast lines represents a significant difference between each group and the group CKN. Error bars indicate ± SD, *n* = 6 (ANOVA, followed by the Tukey’s HSD test, *P < 0.05, ***P < 0.001).

To find out how QS affects the ability of *S. rhizophila* to help plants grow, a 5-week plant-growing experiment was conducted. From the comparison of CKN with other groups in Figures 5A, B, noticeably, the growth of plants was inhibited by saline-alkaline stress. Compared with CK group, W, F, and F + D groups significantly increased fresh plant weight by 54.5, 52.3, and 52.9%, respectively. The *S. rhizophila* WT increased the mean aboveground tissue biomass (Figure 5C) and T-AOC level (Figure 5E) of the plants, but there was no significant difference. There was no significant difference in the chlorophyll content (Figure 5D) of the leaves among the groups. As a whole, the growth promoting ability of *S. rhizophila* WT was more substantial than that of Δ*rpfF.*

No plant growth-promoting effect was observed in SW, SF, and D groups. This result indicates that the active *S. rhizophila* WT and Δ*rpfF* help plants resist saline-alkaline stress, rather than the added organic carbon. In addition, DSF had no growth promoting function on plants, and the growth promoting ability of Δ*rpfF* was not enhanced after the addition of DSF.

## 4. Discussion

Here, we applied bacterial growth monitoring, DSF secretion monitoring, detection of exogenous DSF addition, seed germination testing, and plant physiology testing under saline-alkaline stress conditions.

In the process of extracting DSF in the prior art, methanol is mostly used (Deng et al., 2010; Huedo et al., 2015; Xu et al., 2015; Zhou et al., 2017a), and the methanol is easy to have an esterification reaction with a long-chain fatty acid substance (De Boer and Bahri, 2011), so the detection result is affected. In this study, acetonitrile was used to dissolve and store DSF, and the substance would not react with the solvent, which was helpful to the stable storage of the sample and had high stability.

However, as DSF is a stable fatty acid chain, it is difficult to ionize and generate collisions, and the reaction intensity of distinctive fragment ions is low. Therefore, the “pseudo-molecular” MRM detection mode is used to select the same ion as the parent ion as the daughter ion, and the mass spectrum signal is collected (Schiesel et al., 2010) (Supplementary Figure 1).

Furthermore, this work demonstrated that the *rpfF* gene was the primary enzyme-coding gene controlling DSF production in *S. rhizophila* strain DSM14405^T^, and that the *rpfF* gene-deficient strain was incapable of producing DSF (Figure 1D). By quantitative measurement of DSF during bacterial growth, we determined that the DSF released by *S. rhizophila* WT under saline-alkaline stress was approximately twice that secreted under normal culture conditions (Figure 2B). The findings of the DSF addback experiment indicate that the exogenous addition of DSF greatly boosted the population density of Δ*rpfF*, and that the promotion impact of double addition was much greater than that of a single addition (Figure 3). Adding DSF twice cannot raise the number of cells in the Δ*rpfF* strain to the same level as the WT strain. This data suggests that DSF is detected by receptor protein after entering Δ*rpfF*, and subsequently modulates the metabolic activity of bacteria to aid in their resistance to saline-alkaline stress. After DSF was entirely consumed and decomposed, the QS regulation in Δ*rpfF* was halted, and population expansion was again limited, necessitating further DSF replenishment. This study demonstrates that DSF-QS regulates the development and metabolism of bacteria precisely and in a timely manner to guarantee their survival. The aforementioned findings demonstrated that DSF-QS is the mechanism *via* which *S. rhizophila* resists saline-alkaline stress.

The germination rate of oilseed rape seeds treated with the *S. rhizophila* WT strain was much higher than that of seeds treated with the Δ*rpfF* strain (Figure 4). The mean germination rate rose somewhat in the F and F + D groups. The addition of autoclaved strains reduces the average germination rate of seeds in comparison to the CK group. We hypothesized that the autoclaved strains may be attached to the seed coat as a form of organic carbon, and that the presence of excessive nutrients impeded seed germination (Solberg et al., 2020). The findings of the seed germination experiment demonstrated that the WT had more environmental adaptation and a higher survival rate than the Δ*rpfF* (Figure 4B). The surviving WT might thus encourage seed germination.

Saline-alkaline stress in the soil inhibits plant water absorption during the growth process; fresh weight and biomass are the most direct manifestation of plant growth status (Skiba et al., 2020; Cai et al., 2021). Under saline-alkaline stress, the fresh weight and biomass of plants fell considerably relative to the CKN group (Figures 5B, C). *S. rhizophila* WT, Δ*rpfF* and Δ*rpfF* + DSF were able to considerably improve the fresh weight of plants and assist plants survive saline-alkaline stress when compared to the CK group (Figure 5B). *S. rhizophila* WT increased the average values of plant biomass (Figure 5C) and T-AOC level (Figure 5E). Under normal circumstances, homeostasis maintains the metabolic status of the plant. Yet, in saline-alkaline stress settings, the metabolic equilibrium of plants is continuously disturbed and ultimately altered. By degrading the structure of the cell membrane, saline-alkaline stress disrupts the osmotic balance of the plant cell and causes the accumulation of various harmful ions. In addition, the excessive reactive oxygen species caused by saline-alkaline stress oxidize cellular components, cause oxidative damage to plants, and irreversibly harm their cells (Julkowska and Testerink, 2015). T-AOC reflects the exhaustive measurement of diverse antioxidant enzymes and non-enzymes in plants (Wang et al., 2012). It conveys, to some extent, the resilience of plants to oxidative damage. The amount of T-AOC in the W group was substantially greater than that in the CKN group, indicating that WT significantly boosted the antioxidant capacity of plants (Figure 5E). Both WT and Δ*rpfF* with the same biomass were able to improve the salt tolerance of plants, as demonstrated by the data presented above (Figure 5B).

Because of its greater environmental flexibility than Δ*rpfF*, WT can increase the germination rate of seeds under saline-alkaline stress. DSF did not directly enhance plants’ resistance to saline-alkaline stress, nor could it enhance the growth-promoting properties of Δ*rpfF*.

Previous studies have demonstrated that *S. rhizophila* produces highly effective osmoprotectants, such as GG, trehalose, and spermidine, which act directly upon roots and aid plant survival in punitive environmental conditions (Alavi et al., 2013b; Egamberdieva et al., 2016). Alavi et al. (2013a) carried out a 5-day incubation experiment of plant seedlings under 0.85% NaCl stress. The fresh weight of the underground parts was weighed, and it was found that the fresh weight of the *S maltophilia* WT treated plants was significantly higher than that of the *S maltophilia* Δ*rpfF*. Therefore, the authors believed that DSF positively affects the early stages of plant growth in *S maltophilia*.

In this study, however, we demonstrate that DSF promotes plant development indirectly rather than directly. DSF enables bacteria to directly resist stress and increase their survival rate, allowing active PGPR to stimulate plant development. Experiments using the anaplerotic addition of DSF indicated that the function of DSF to aid *S. rhizophila* in resisting stress on their own is time-efficient. The addition of DSF to the Δ*rpfF* did not boost the seed germination rate to the level of the WT, nor did it increase the growth-promoting ability of the Δ*rpfF*, demonstrating that the addition of DSF alone cannot alter the function of bacteria. Continuous and intricate regulation of DSF in bacterial populations was observed.

*S. rhizophila* WT boosted their survival rate to ensure a sufficient bacterial population by increasing the release of DSF under saline-alkaline stress. Furthermore, *S. rhizophila* WT had a growth-promoting role in promoting seed germination. But, under stress, Δ*rpfF*’s growth was slowed, its population fell, and its growth-promoting impact was naturally constrained. In addition, DSF has no direct influence on the growth of plants. We revealed that the DSF-QS system has a direct favorable influence on the environmental adaption of *S. rhizophila*, hence indirectly enhancing the growth-promoting capacity of the WT.

## 5. Conclusion

This study established a novel method for the quantitative determination of DSF by UHPLC-MS/MS using the “pseudo-molecular” MRM detection mode. We constructed the *rpfF*-knockout mutant strain that was unable to secrete DSF. The DSF-QS system aims to regulate the growth of bacteria themselves, double-secreting DSF under saline-alkaline stress, promoting the bacterial population by improving their environmental adaptability and survival rate, thereby enabling PGPR to exert its growth-promoting effect. In addition, DSF cannot directly help plants resist saline-alkali stress. This study deepens our understanding of DSF-QS survival and growth-promoting strategies in PGPR.

## Data availability statement

The original contributions presented in this study are included in the article/Supplementary material, further inquiries can be directed to the corresponding author.

## Author contributions

XZ: conceptualization, methodology, supervision, project administration, funding acquisition, data curation, formal analysis, and writing—review and editing. YL: investigation, data curation, formal analysis, validation, visualization, and writing—original draft. NF: investigation, software, formal analysis, validation, and visualization. ZB: methodology, resources, and writing—review and editing. JG: conceptualization, methodology, resources, data curation, formal analysis, and writing—review and editing. All authors contributed to the article and approved the submitted version.

## Footnotes

1
https://blast.ncbi.nlm.nih.gov/Blast.cgi

2
https://www.ncbi.nlm.nih.gov/

3
https://www.ebi.ac.uk/Tools/msa/clustalo/

## References

[B1] Alavi P. Starcher M. R. Thallinger G. G. Zachow C. Mueller H. Berg G. (2014). *Stenotrophomonas* comparative genomics reveals genes and functions that differentiate beneficial and pathogenic bacteria. *BMC Genom.* 15:482. 10.1186/1471-2164-15-482 24939220 PMC4101175

[B2] Alavi P. Starcher M. R. Zachow C. Muller H. Berg G. (2013b). Root-microbe systems: The effect and mode of interaction of stress protecting agent (SPA) *Stenotrophomonas rhizophila* DSM14405(T). *Front. Plant Sci.* 4:141. 10.3389/fpls.2013.00141 23717321 PMC3653106

[B3] Alavi P. Muller H. Cardinale M. Zachow C. Sanchez M. B. Martinez J. L. (2013a). The DSF quorum sensing system controls the positive influence of *Stenotrophomonas maltophilia* on plants. *PLoS One* 8:e67103. 10.1371/journal.pone.0067103 23874407 PMC3715506

[B4] Cai Z. Liu X. Chen H. Yang R. Chen J. Zou L. (2021). Variations in morphology, physiology, and multiple bioactive constituents of Lonicerae Japonicae Flos under salt stress. *Sci. Rep.* 11 1–15. 10.1038/s41598-021-83566-6 33594134 PMC7887249

[B5] Calatrava-Morales N. McIntosh M. Soto M. J. (2018). Regulation mediated by N-Acyl homoserine lactone quorum sensing signals in the rhizobium-legume symbiosis. *Genes* 9:263. 10.3390/genes9050263 29783703 PMC5977203

[B6] De Boer K. Bahri P. A. (2011). Supercritical methanol for fatty acid methyl ester production: A review. *Biomass Bioenergy* 35 983–991.

[B7] Deng Y. Wu J.e Eberl L. Zhang L.-H. (2010). Structural and functional characterization of diffusible signal factor family quorum-sensing signals produced by members of the *Burkholderia cepacia* complex. *Appl. Environ. Microbiol.* 76 4675–4683. 10.1128/AEM.00480-10 20511428 PMC2901730

[B8] Egamberdieva D. Jabborova D. Berg G. (2016). Synergistic interactions between Bradyrhizobium japonicum and the endophyte *Stenotrophomonas rhizophila* and their effects on growth, and nodulation of soybean under salt stress. *Plant Soil* 405 35–45. 10.1007/s11104-015-2661-8

[B9] He Y. W. Wu J. E. Cha J. S. Zhang L. H. (2010). Rice bacterial blight pathogen *Xanthomonas oryzae* pv. oryzae produces multiple DSF-family signals in regulation of virulence factor production. *BMC Microbiol.* 10:187. 10.1186/1471-2180-10-187 20615263 PMC2909994

[B10] Hu L. Robert C. A. M. Cadot S. Zhang X. Ye M. Li B. (2018). Root exudate metabolites drive plant-soil feedbacks on growth and defense by shaping the rhizosphere microbiota. *Nat. Commun.* 9:2738. 10.1038/s41467-018-05122-7 30013066 PMC6048113

[B11] Huang T. P. Lee Wong A. C. (2007). Extracellular fatty acids facilitate flagella-independent translocation by *Stenotrophomonas maltophilia*. *Res. Microbiol.* 158 702–711. 10.1016/j.resmic.2007.09.002 18054205

[B12] Huedo P. Yero D. Martinez-Servat S. Ruyra A. Roher N. Daura X. (2015). Decoding the genetic and functional diversity of the DSF quorum-sensing system in *Stenotrophomonas maltophilia*. *Front. Microbiol.* 6:761. 10.3389/fmicb.2015.00761 26284046 PMC4517397

[B13] Julkowska M. M. Testerink C. (2015). Tuning plant signaling and growth to survive salt. *Trends Plant Sci.* 20 586–594. 26205171 10.1016/j.tplants.2015.06.008

[B14] Kumar A. Verma J. P. (2018). Does plant-microbe interaction confer stress tolerance in plants: A review? *Microbiol. Res.* 207 41–52. 10.1016/j.micres.2017.11.004 29458867

[B15] Li L. Li J. Zhang Y. Wang N. (2019). Diffusible signal factor (DSF)-mediated quorum sensing modulates expression of diverse traits in *Xanthomonas citri* and responses of citrus plants to promote disease. *BMC Genom.* 20:55. 10.1186/s12864-018-5384-4 30654743 PMC6337780

[B16] Li Q. Yang A. Zhang W. H. (2016). Efficient acquisition of iron confers greater tolerance to saline-alkaline stress in rice (*Oryza sativa* L.). *J. Exp. Bot.* 67 6431–6444. 10.1093/jxb/erw407 27811002 PMC5181582

[B17] Lian T. Huang Y. Xie X. Huo X. Shahid M. Q. Tian L. (2020). Rice SST variation shapes the rhizosphere bacterial community, conferring tolerance to salt stress through regulating soil metabolites. *mSystems* 5 e721–e720. 10.1128/mSystems.00721-20 33234605 PMC7687028

[B18] Liu J. Shen F. Xiao Y. Fang H. Qiu C. Li W. (2020). Genomics-assisted prediction of salt and alkali tolerances and functional marker development in apple rootstocks. *BMC Genom.* 21:550. 10.1186/s12864-020-06961-9 32778069 PMC7430842

[B19] Liu Y. Gao J. Wang N. Li X. Fang N. Zhuang X. (2022). Diffusible signal factor enhances the saline-alkaline resistance and rhizosphere colonization of *Stenotrophomonas rhizophila* by coordinating optimal metabolism. *Sci. Total Environ.* 834:155403. 10.1016/j.scitotenv.2022.155403 35469877

[B20] Paul D. Lade H. (2014). Plant-growth-promoting rhizobacteria to improve crop growth in saline soils: A review. *Agron. Sustain. Dev.* 34 737–752. 10.1007/s13593-014-0233-6

[B21] Phour M. Sehrawat A. Sindhu S. S. Glick B. R. (2020). Interkingdom signaling in plant-rhizomicrobiome interactions for sustainable agriculture. *Microbiol. Res.* 241:126589. 10.1016/j.micres.2020.126589 32927204

[B22] Ryan R. P. An S. Q. Allan J. H. McCarthy Y. Dow J. M. (2015). The DSF family of cell-cell signals: An expanding class of bacterial virulence regulators. *PLoS Pathog.* 11:e1004986. 10.1371/journal.ppat.1004986 26181439 PMC4504480

[B23] Schiesel S. Laemmerhofer M. Lindner W. (2010). Quantitative LC-ESI-MS/MS metabolic profiling method for fatty acids and lipophilic metabolites in fermentation broths from beta-lactam antibiotics production. *Anal. Bioanal. Chem.* 397 147–160. 10.1007/s00216-009-3340-5 20024533

[B24] Skiba E. Pietrzak M. Gapińska M. Wolf W. M. (2020). Metal homeostasis and gas exchange dynamics in *Pisum sativum* L. exposed to cerium oxide nanoparticles. *Int. J. Mol. Sci.* 21:8497. 10.3390/ijms21228497 33187383 PMC7696629

[B25] Solberg S. Ø Yndgaard F. Andreasen C. von Bothmer R. Loskutov I. G. Asdal Å (2020). Long-term storage and longevity of orthodox seeds: A systematic review. *Front. Plant Sci.* 11:1007. 10.3389/fpls.2020.01007 32719707 PMC7347988

[B26] Wang G. Zhu Q. Meng Q. Wu C. (2012). Transcript profiling during salt stress of young cotton (*Gossypium hirsutum*) seedlings via Solexa sequencing. *Acta Physiol. Plant.* 34 107–115.

[B27] Wang L. H. He Y. Gao Y. Wu J. E. Dong Y. H. He C. (2004). A bacterial cell-cell communication signal with cross-kingdom structural analogues. *Mol. Microbiol.* 51 903–912. 10.1046/j.1365-2958.2003.03883.x 14731288

[B28] Xu J. Zhou L. Venturi V. He Y.-W. Kojima M. Sakakibari H. (2015). Phytohormone-mediated interkingdom signaling shapes the outcome of rice-*Xanthomonas oryzae* pv. oryzae interactions. *BMC Plant Biol.* 15:10. 10.1186/s12870-014-0411-3 25605284 PMC4307914

[B29] Xu X. X. Zhang J. J. Yan B. W. Wei Y. L. Ge S. N. Li J. X. (2021). The adjustment of membrane lipid metabolism pathways in maize roots under saline-alkaline stress. *Front. Plant Sci.* 12:635327. 10.3389/fpls.2021.635327 33790924 PMC8006331

[B30] Zhou L. Zhang L. H. Camara M. He Y. W. (2017b). The DSF family of quorum sensing signals: Diversity, biosynthesis, and turnover. *Trends Microbiol.* 25 293–303. 10.1016/j.tim.2016.11.013 27979499

[B31] Zhou L. Wang X.-Y. Zhang W. Sun S. He Y.-W. (2017a). Extraction, purification and quantification of diffusible signal factor family quorum-sensing signal molecules in *Xanthomonas oryzae* pv. oryzae. *Bioprotocol* 7 e2190–e2190. 10.21769/BioProtoc.2190 34458499 PMC8376614

