# Supplementary material for: Quorum sensing improves the plant growth-promoting ability of Stenotrophomonas rhizophila under saline-alkaline stress by enhancing its environmental adaptability
Source: Front Microbiol. 2023 Apr 11;14:1155081. doi: 10.3389/fmicb.2023.1155081 (PMC10126360; doi:10.3389/fmicb.2023.1155081)
Supplement: Supplementary file 1 [file Data_Sheet_1.pdf]

SUPPLEMENTARY INFORMATION

DSF-quorum sensing improves the plant growth-promoting ability of *Stenotrophomonas rhizophila* under saline-alkaline stress by enhancing its environmental adaptability

Xuliang Zhuang[1,2,3], Ying Liu[1,4], Na Fang[1,5], Zhihui Bai[1,2,6] and Jie Gao[1,2*]

[1]CAS Key Laboratory of Environmental Biotechnology, Research Center for Eco-Environmental Sciences, Chinese Academy of Sciences, Beijing 100085, China;
[2]College of Resources and Environment, University of Chinese Academy of Sciences, Beijing 100049, China;
[3]State Key Laboratory of Tibetan Plateau Earth System, Environment and Resources (TPESER), Institute of Tibetan Plateau Research, Chinese Academy of Sciences, Beijing 100101, China;
[4]Peking University Institute of Advanced Agricultural Sciences, Weifang 261325, China;
[5]Institute of International Rivers and Eco-security, Yunan University, Kunming 650500, China;
[6]Xiongan Institute of Innovation, Xiongan New Area, 071800, China.

*** Corresponding author:**
Email: jiegao@rcees.ac.cn (Jie Gao)
P.O.Box 2871, Beijing, China 100085
Tel. & Fax: +86-10-62923562

The following Supporting Information is available for this article:

Table S1 Primer sequences of *S. rhizophila* Δ*rpfF* construction.

Table S2 Soil parameter.

Fig. S1 Detection of DSF.

Table S1 Primer sequences of *S. rhizophila* Δ*rpfF* construction.

Primer name	Primer sequence
rpfF-MF1	GGAATCTAGACCTTGAGTCGTGTCCACTCCTTGAACGGTCTATT
rpfF-MR1	ATCCGCATCATTTCGTGCAGGTGCGCTTCTTCGGTGATGC
rpfF-MF2	GCATCACCGAAGAAGCGCACCTGCACGAAATGATGCGGAT
rpfF-MR2	ACAGCTAGCGACGATATGTCGGGAGGGGTTTGAAAAGCAGT
rpfF -UF	GACACAGTTGTAACTGGTCCA
rpfF -UR	CAGGAACACTTAACGGCTGAC
rpfF-TF	TCCACCCAGAAGTGCGATGA
rpfF-TR	GGTGCTCAGTGCCGATGTCA

Table S2 Soil parameter.

Soil parameter	Value
pH	8.02±0.31
Electrical conductivity (μS/cm)	718.03±24.89
Organic carbon content (%)	0.71±0.08
Total carbon content ($g.kg^{-1}$)	20.8±0.27
Total nitrogen ($g.kg^{-1}$)	1.41±0.04
Total S concentration ($g.kg^{-1}$)	0.38±0.05
Total Al concentration ($mg.g^{-1}$)	1043.76±7.01
Total Ca concentration ($mg.g^{-1}$)	2918.67±18.66
Total Fe concentration ($mg.g^{-1}$)	2003±19.61
Total K concentration ($mg.g^{-1}$)	1769±28.90
Total Mg concentration ($mg.g^{-1}$)	561.89±10.39
Total Mn concentration ($mg.g^{-1}$)	49.97±6.34
Total Na concentration ($mg.g^{-1}$)	1735.91±19.22
Total P concentration ($mg.g^{-1}$)	273.04±11.50

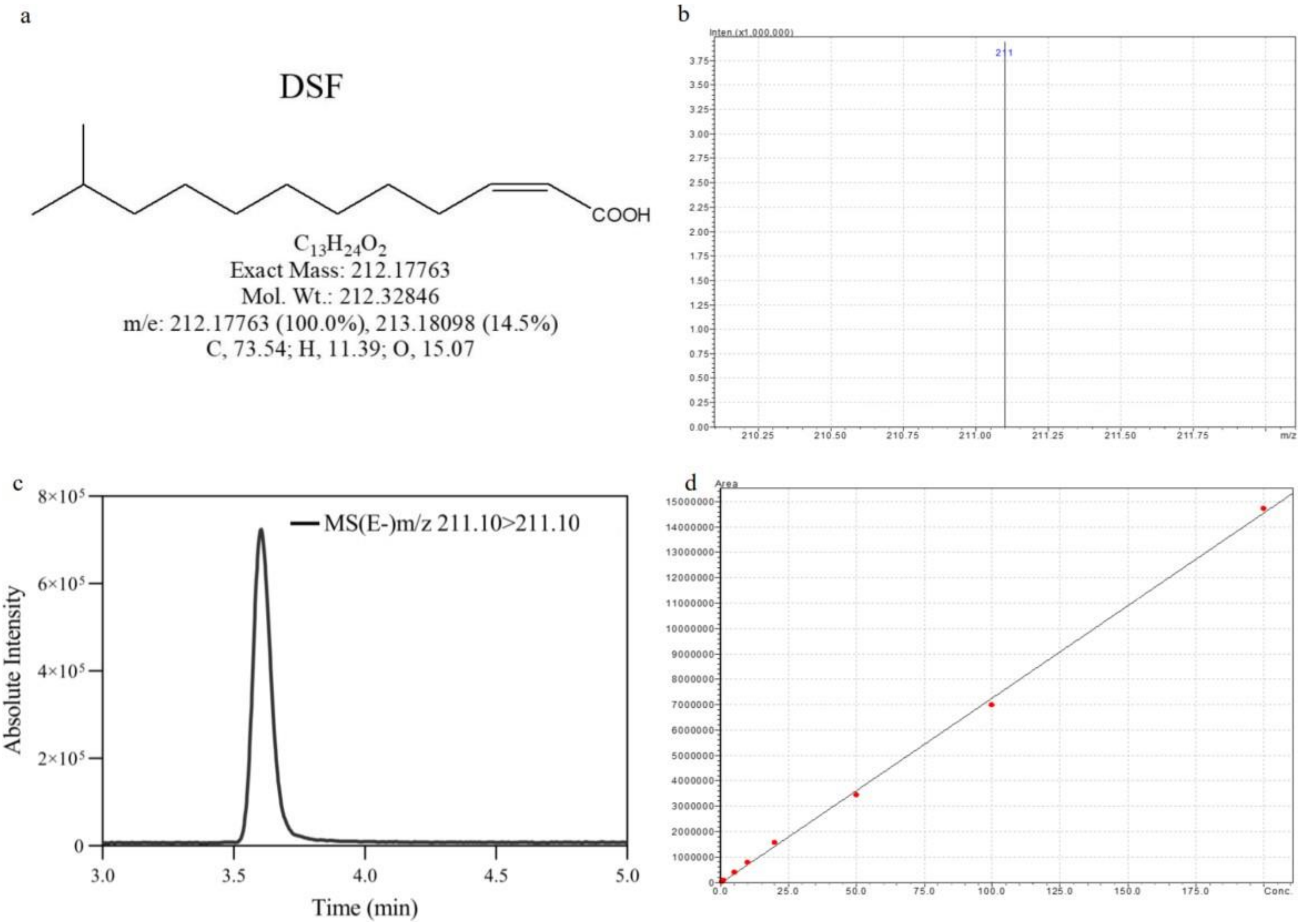

Fig S1 (a) The structural formula of DSF. (b) Secondary ion mass spectrometry diagram of DSF. (c) Representative MRM (multiple-reaction monitoring) chromatogram of DSF peak in *S. rhizophila* WT. (d) DSF standard curve.
